# Tension Reduction With Force Modulating Tissue Bridges Reduces Wounds in Breast Surgery

**DOI:** 10.1093/asj/sjad285

**Published:** 2023-08-31

**Authors:** Holly C Wall, Sameer H Halani, Chizoba Mosieri, Charles Daniel, Lucas Gallo, Felmont F Eaves, Christopher J Coroneos

## Abstract

**Background:**

Tension on healing wounds increases the risk of dehiscence and poor or pathologic scar formation. Force modulating tissue bridges (FMTBs) represent a new class of wound closure and support devices designed to offload tension on healing wounds to improve wound healing and scar outcomes.

**Objectives:**

The study was undertaken to assess the efficacy of FMTBs to reduce the risk of wound healing complications in elective breast surgery.

**Methods:**

One hundred twenty-two consecutive patients undergoing bilateral aesthetic breast surgery underwent intraoperative placement of FMTBs on the vertical limb closure site. A matched case–control cohort of 121 consecutive patients was established for comparison. Wounds were considered significant if larger than 3 mm in diameter. The primary outcome of breast wounds >3 mm was reported with a relative risk, and all outcomes were framed with number needed to treat.

**Results:**

The control and intervention cohorts had similar demographics, comorbidities, type of operation, and incision pattern utilized. Within the FMTB group, 96.7% (*n* = 118) patients completed treatment per protocol. Significant wounds occurred in 1.7% (*n* = 2) of patients in the tissue bridge vs 15.2% (*n* = 19) in controls on a per patient/per protocol basis (89% reduction, *P* < .001). Statistically significant improvements were maintained on sensitivity analyses with intention to treat, even when minor wounds were included. There were no complications noted related to FMTBs.

**Conclusions:**

FMTBs are safe and highly effective at reducing the risk of wound formation in elective breast surgery. Results are consistent with sensitivity analyses based on clinical and methodological factors. Further research will assess long-term scar outcomes.

**Level of Evidence: 4:**

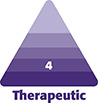

Wound complications in breast surgery can be a significant issue leading to patient anxiety, reduced patient satisfaction, additional treatment requirements, impaired aesthetic outcomes, and additional surgery including scar revision.^1-4^ Furthermore, when associated with breast reconstruction, wound complications can lead to a delay in adjunctive oncologic therapy.^5^ A loss of closure integrity, variously described as wound dehiscence, delayed wound healing, wound separation, or an open wound, is perhaps the most common complication in benign elective breast surgeries such as mastopexy, augmentation-mastopexy, and reduction mammaplasty, with reported rates exceeding 20% in some studies.^6-8^ Although often minor, wound dehiscence can be associated with more severe complications such as localized soft tissue infection, poor scar outcomes, and threatened or actual exposure of prosthetic materials including breast implants or support materials such as synthetic or biologic mesh.

It is hard to adequately describe the impact small wounds have on patient psyche, the surgeon, and staff morale. Even modest, “eraser size” wounds typically take weeks to heal, and larger ones longer still due to tension and location of the opening. This has an enormous negative impact on the postoperative course for patients, surgeon, and staff. For patients who suffer wound separation requiring intervention, it is difficult to quantify the increased level of anxiety and dissatisfaction these patients experience, although their reviews are quite explicit and may be multiple. Because elective breast surgery is extremely common in plastic surgery, with more than 167,000 US and 781,000 global mastopexies and 82,000 US and 500,000 global breast reductions performed in 2021, decreasing dehiscence in breast surgery can impact a large number of patients’ and surgeons’ quality of life.^9,10^

Multiple patient-related factors increase the risk of wound dehiscence: obesity, smoking, diabetes, impaired nutritional status, history of massive weight loss, previous irradiation, and poor tissue quality.^1,11-14^ Medications including chemotherapeutic agents, steroids, and other immunologic treatments also predispose to dehiscence.^15,16^ Wound tension is a significant, ubiquitous risk factor which can be further potentiated by tissue ischemia and swelling. Wound closure technique and forces imparted by sutures can reduce perfusion to the healing wound and lead to focal necrosis.^17-19^ Reducing tissue excision, avoiding pleating, and limitation of positional changes have proven effective in reducing wound complications of dehiscence in body contouring.^11,20^ Closed-incision negative-pressure wound therapy may reduce dehiscence due to changes in wound forces, and tension reduction has also been shown effective in improvement of scar outcomes, as seen in devices such as Embrace (Neodyne Biosciences, Inc., Fremont, CA).^21-25^

Force Modulating Tissue Bridges (FMTB or “tissue bridges,” Brijjit; Brijjit Medical, Inc., Marietta, GA) are a new type of medical device designed to offload cutaneous wound tension. In superficial wounds FMTBs can be applied as a single-layer definitive wound closure. In this study, FMTBs were applied over the final suture layer in elective cosmetic breast surgery patients to provide immediate wound support by tension offloading with a goal of adherence for 3 to 6 weeks. In strain mapping studies, FMTBs have shown high efficiency in cutaneous tension reduction, and tension mitigation is maintained as long as the device is adherent.^26^ The primary objective of this study was to analyze the efficacy of FMTBs in reducing wounds in breast surgery when placed over traditional closure with absorbable sutures.

## METHODS

This was a retrospective cohort study. Beginning in December 2022, 122 consecutive elective female breast surgery patients underwent placement of FMTBs to the vertical incision closure site of each breast by H.C.W. Male patients were excluded from this study. All augmentation-mastopexy, mastopexy, mastopexy with implant exchange, and reduction mammaplasty surgeries were included. A paired cohort of consecutive patients with corresponding procedures treated before the adoption of FMTBs as part of the surgical protocol were identified as the control group. The Ethical Principles for Medical Research from the World Medical Association Declaration of Helsinki Involving Human Subjects were observed in the completion of this study. All patients provided written informed consent prior to undergoing surgery.

Except for the routine incorporation of FMTBs, neither the author's surgical techniques nor materials varied during treatment of the control and FMTB cohorts. All patients underwent a superiorly based pedicle technique with a circumvertical incision pattern with or without a horizontal excision (inverted T or lateral J) to address the excess caudal skin. Some patients also underwent placement of a subpectoral breast implant with the author's described technique.^27^ Final closures of the vertical and horizontal limbs were performed by placement of inverted interrupted deep dermal sutures of 3-0 poliglecaprone 25 (Monocryl; Ethicon, Inc., Bridgewater, NJ) followed by running suture of the same material. No external sutures were placed for additional support.

**Figure 1.**
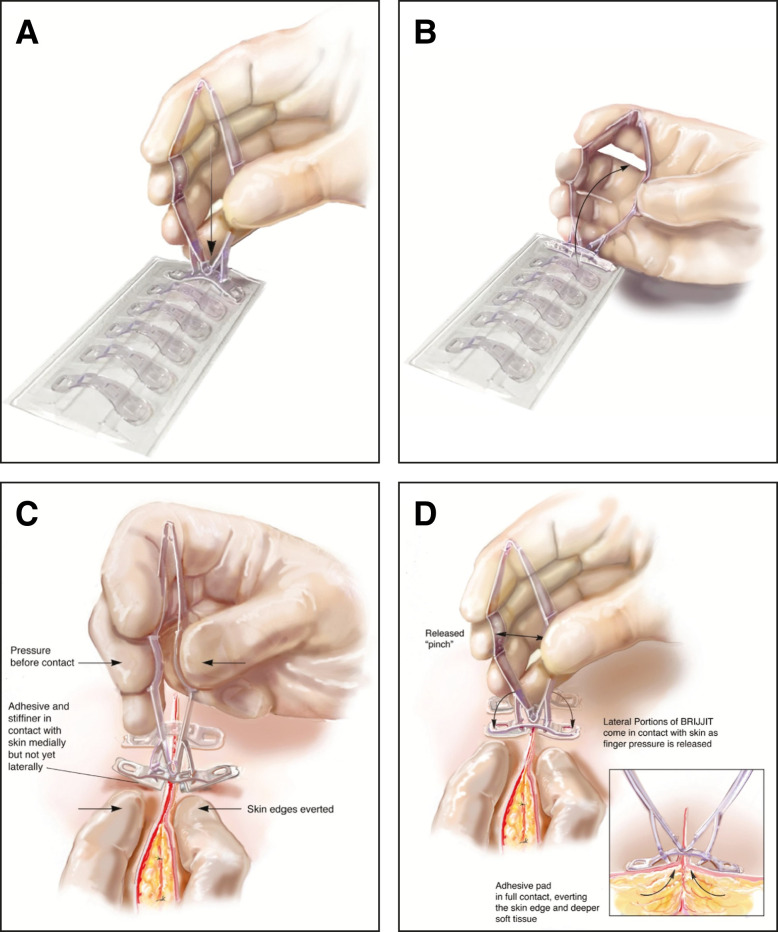
(A) The force modulating tissue bridge (FMTB) is loaded onto the applicator by inserting the catch arms into the central windows. The applicator is not compressed during the loading process. (B) To begin release of the FMTB from the loading tray, the attached applicator is pivoted toward either end of the loading tray. (C) To apply, the wound is held in approximation either with the fingers, as shown, or with forceps. The applicator is compressed, bending (preloading) the FMTB, and the preloaded FMTB is centered over the wound and lowered. The medial struts will be the first points of contact on either side. (D) With gentle pressure applied, the applicator compression is relaxed, allowing the lateral sections to rotate into position. Inset: Relaxation of the applicator produces a rotational movement with downward lateral force, combined with a medial movement, to produce an upward and inward force vector at the wound interface. These transmitted rotational forces produce eversion of the wound and mitigate tension of the superficial tissues. The applicator is now ready for removal. Figures produced by Bill Winn and reprinted with permission from BRIJJITCO, LLC/Brijjit Medical, Inc. Reprinted with permission from D. O. Kazmer and F. F. Eaves.^26^

**Figure 2.**
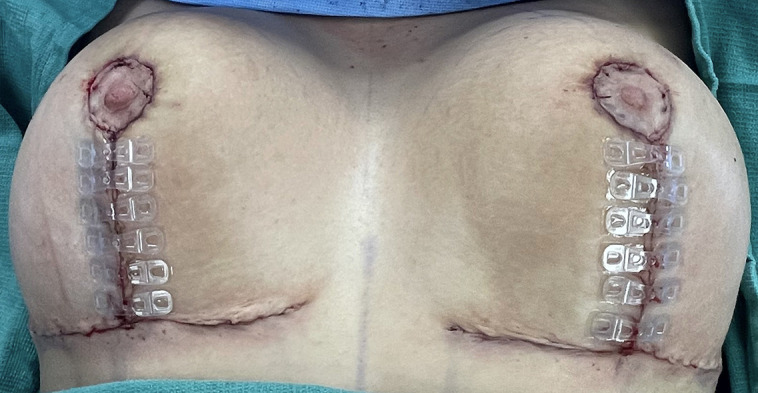
A 16-year-old female patient undergoing aesthetic breast reduction with tissue bridges in place at the end of the procedure, before placement of dressings.

At completion of the procedure, skin preparation for tissue bridge placement consisted of degreasing the skin on either side of the incision with a standard alcohol pad. FMTBs were removed from the loading tray by attachment to and rotation of the supplied applicator, exposing the patient contact adhesive (Figure 1A). Before placing each FMTB over the incision, the applicator was squeezed to produce preapplication deformational loading (Figure 1B). The medial struts of the device contacted the skin first and were pressed against the wound margin by the applicator pins to facilitate initial adherence (Figure 1C). As the applicator was relaxed, the lateral part of the device rotated into contact with the skin on each side, producing central advancement of the underlying tissue (Figure 1D). FMTBs were placed over the vertical incision, including immediately above the lower T-junction locations, in series and spaced per the manufacturer's recommendation (Video; Figure 2). The adhesive for this device was made of a medical grade acrylic. A fanfold gauze was placed over the breast and secured with tape. Tape was not applied over the FMTBs to avoid accidental detachment with dressing removal. Self-adhering foam dressings (Reston; 3 M, Maplewood MN) were placed directly caudal to the breast and a postsurgical bra was placed in the recovery room. Patients were allowed to shower 48 hours after surgery and replace the foam and bra. The FMTBs were not actively removed, but rather were allowed to detach passively. In this study, any devices that failed or were removed prematurely (<1 week) were not replaced.

Patient charts were reviewed for demographic information, including age, race, body mass index (BMI), diabetes, smoking status, and previous breast surgery, as well as for procedure-specific information including pedicle type, tissue resection amount, implant information, and concurrent procedures. Per protocol treatment consisted of device adherence for a minimum of 1 week. The primary outcome of a breast wound dehiscence was defined as a delayed open wound 3 mm or greater in size occurring in the vertical limb closure area as measured by the operating surgeon. Wounds remote to the treatment area (eg, upper aspect of the areola) were not included in the analysis. Wounds were noted as unilateral or bilateral. Charts were reviewed to identify if any blistering, infection, hematoma, seroma, nipple loss, or other complications occurred.

To analyze the primary outcome, descriptive statistics (ie, percentages, means, standard deviations) were reported to determine the incidence of breast wound dehiscence. Continuous variables were evaluated with an independent *t* test. Categorical variables were analyzed with a chi-square test and Fischer's exact test. The primary outcome of breast wound dehiscence was reported with a relative risk (RR) with 95% confidence interval. The primary outcome was reported per protocol. Sensitivity analyses were reported with intention to treat (ITT) and included wounds less than 3 mm. All outcomes were framed with number needed to treat (NNT). Pairwise deletion (ie, available case analysis) addressed missing data within the analysis. Statistical significance was set at *P* ≤ .05. All analyses were performed with SPSS 26.0 (IBM, Armonk, NY).

## RESULTS

Two hundred forty-three consecutive female patients were included in our study who underwent either breast reductions (*n* = 33, 13.6%); mastopexy (*n* = 31, 12.8%); mastopexy-augmentation (*n* = 176, 72.4%); or mastopexy with implant exchange (*n* = 3, 1.2%). Among these, 121 (49.7%) had conventional closure methods and 122 (50.2%) utilized the FMTBs. The mean age in the FMTB group was 39.8 ± 10.6 years vs 36.1 ± 10.6 years in the conventional group (*P* = .97). The key demographic details are available in Table 1; there were no significant differences in demographic data between the 2 groups, including a comparable BMI, number of smokers, and number of diabetics.

**Table 1.** Patient Characteristics

Participant characteristics		FMTB		Control		*P*
		*n* = 122	%	*n* = 121	%	
Age, years	Mean (SD)	121	39.8 (10.6)	120	36.1 (10.6)	.97
BMI	Mean (SD)	122	26.2 (3.6)	121	25.7 (3.4)	.17
Ethnicity	Caucasian	117	95.9	114	94.2	.71
	Hispanic	4	3.3	4	3.3	
	African American	1	0.8	2	1.7	
	Asian	0	0	1	0.8	
Diabetes status	Yes	0	0	3	2.5	.08
	No	122	100	118	97.5	
Smoking status	Yes	13	10.7	14	11.6	.87
	No	109	89.3	107	88.4	
Previous breast surgery	Yes	36	29.5	14	88.4	.01*
	No	86	70.5	107	11.6	
Massive weight loss	Yes	10	8.2	7	94.2	.46
	No	112	91.8	114	5.8	
Type of procedure	Mastopexy	16	13.1	15	12.4	.38
	Mastopexy & implant exchange	3	2.5	0	0	
	Mastopexy & augmentation	87	71.3	89	73.6	
	Breast reduction	16	13.1	17	14.0	
Incision pattern	Vertical pattern	53	43.4	46	38.0	.44
	Wise pattern	69	56.6	74	61.2	
	Missing	0	0	1	0.8	
Breast wound dehiscence	Yes	7	5.7	17	14.0	.03*
	No	115	94.3	104	86.0	
Incision site infection	Yes	5	4.1	8	6.6	.38
	No	117	95.9	113	93.4	
Breast hematoma	Yes	2	1.6	1	0.8	.56
	No	120	98.4	120	99.2	
Breast seroma	Yes	9	7.4	7	5.8	.62
	No	113	92.6	114	94.2	

*P* value was obtained based on chi-square test or Fisher's exact test as appropriate. *Indicates statistically significant value (*P* < .05). BMI, body mass index; FMTB, force modulating tissue bridges; SD, standard deviation.

The median postoperative follow-up was 5.33 months (range 0.27 to 50.4 months) in the conventional group and 3.53 months (range 0.5 to 20.3 months) in the FMTB group. Within the conventional group, 89 (73.6%) patients underwent single-stage augmentation-mastopexy, 17 (14.0%) underwent breast reductions, and 15 (12.4%) underwent mastopexies; in the FMTB group, the split was comparable, with 87 (71.3%) patients undergoing single-stage augmentation-mastopexy, 16 (13.1%) breast reduction, 16 (13.1%) mastopexies, and 3 (2.5%) implant exchange with mastopexy. There was no significant difference in the distribution of vertical only and vertical plus horizontal incisions between the groups (*P* = .52). Within the FMTB group, 4 patients had premature removal or detachment of the device; 3 of whom had the device fall off within the first week of placement, and 1 erroneously removed it on her own. Premature device detachments occurred early in the period of device adoption in the practice, illustrating a learning curve of skin preparation, because these were likely attributable to inadequate degreasing of the skin before application. No premature device detachments occurred after these 4 patients.

**Figure 3.**
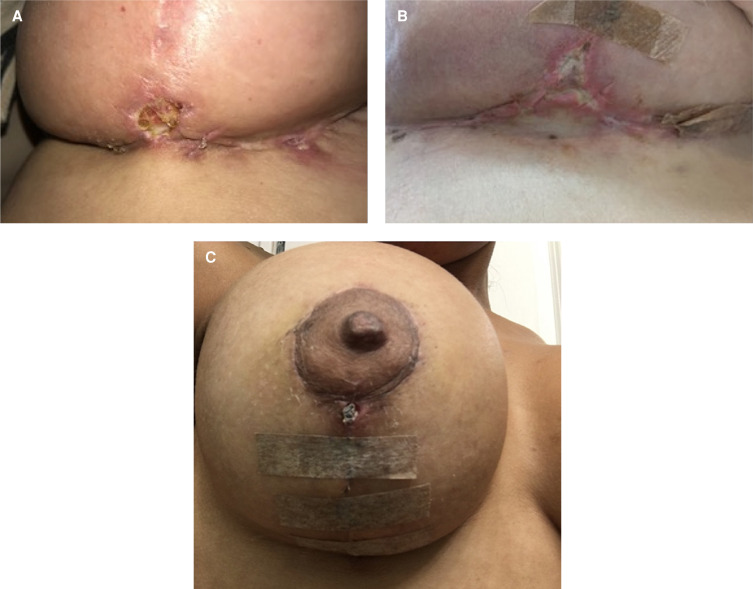
(A) Example of a significant inverted T junction breakdown in a 32-year-old female patient who has undergone augmentation-mastopexy without the use of FMTBs. Open wounds in the presence of an underlying implant enhance the risk of implant complications. (B) A 21-year-old female patient with an inverted T wound after mastopexy without the use of FMTBs. (C) Although most commonly open wounds in elective breast surgery occur at the inferior aspect of the vertical incision—with or without a horizontal incision—they may also occur at the upper T junction where the vertical incision intersects with the areola. This 31-year-old female patient underwent wound support with sterile tapes but did not have placement of tissue bridges.

**Figure 4.**
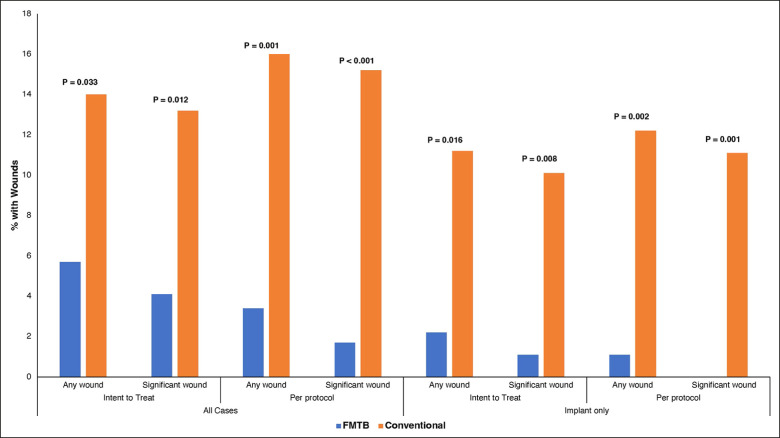
Percentage of patients with open wounds in the control vs force modulating tissue bridge groups by intention to treat and per protocol, separated by significant (>3 mm) and all wounds (including wounds 3 mm or less), and by the presence of implants or no implants. Significance was calculated based on *t* test.

The overall occurrence rate of significant wounds (>3 mm) was 8.6% (*n* = 21) (Figure 3A-C). Analyzed per protocol, there was significant reduction in wounds >3 mm with FMTBs (*n* = 2, 1.7%) vs conventional closure (*n* = 19, 15.2%), *P* < .0001 (RR = 0.11 [95% CI 0.026 to 0.468]). There was an 89% reduction in the relative risk of a significant wound. NNT was 7.40, meaning FMTB on only 8 patients prevented 1 event. Results were consistent when analyzed with ITT, with a significant reduction in wounds for FMTB (4.1%, *n* = 5) compared to the conventional group (13.2%, *n* = 16), *P* = .012 (RR = 0.31 [95% CI 0.117 to 0.819], NNT = 10.95). Sensitivity analyses were repeated with smaller wounds; there were 3 “pinhole” wounds (<3 mm) overall (1 in the control group, 2 in the FMTB group). When including these minor wounds, outcomes for FMTB remained significant by both per protocol and ITT analysis. These results are summarized in Table 2 and Figure 4.

**Table 2.** Risk Reduction Models for FMTB and Conventional Wound Closure Methods

	NNT	RR	95% CI	*P* value
Any breast wound^a^	12.03	0.41	0.175-0.949	.033
Significant wounds^a^	10.95	0.309	0.117-0.819	.012
Any breast wound^b^	7.93	0.21	0.074-0.602	.001
Significant wounds^b^	7.40	0.11	0.026-0.468	<.0001
Implant-based operations				
Any breast wound^a^	11.0	0.19	0.043-0.849	.016
Significant wounds^a^	11.06	0.11	0.014-0.822	.008
Any breast wound^b^	8.98	0.09	0.012-0.674	.002
Significant wounds^b^	9.09	0.05	0.003-7.83	.001

^a^Intention to treat model. ^b^Per protocol model. CI, confidence interval; FMTB, force modulating tissue bridges; NNT, number needed to treat; RR, relative risk of FMTB group to the conventional group.

In subgroup analysis of patients who had procedures with breast implants, there was a significant reduction in major breast wounds in the FMTB cohort (*P* < .001, RR 0.05 (95% CI 0.003 to 0.783). The NNT in this model was 9. Results were consistent with ITT, with NNT of 11. Additional subgroups were modeled; FMTB remained statistically significant after adjusting for presence of an implant vs no implant, but there was no difference between the 2 groups. Further, the FMTB group remained statistically significant after adjusting for vertical only vs vertical plus horizontal incisions, but there was no difference between incision types. There were no FMTB footplate blisters, and no difference in other complications between the control and FMTB groups (Table 1).

## DISCUSSION

FMTBs significantly reduce major breast wounds following elective benign breast surgery. This is a large effect; FMTB demonstrates an 89% relative risk reduction vs conventional closure. Only 8 patients need to be treated with FMTB to prevent a major event. Results are consistent with sensitivity analyses adjusting for multiple clinical and methodological factors. FMTB use is safe, with limited premature device failure.

The implications of a wound are amplified in the setting of an underlying prosthetic implant. FMTB significantly reduces wounds in single-stage augmentation-mastopexies, with even ITT analysis demonstrating a relative risk reduction of 81% and NNT of 12. If FMTB is applied at the time of augmentation-mastopexy, use on 12 patients will prevent 1 major event, even when premature device failures are included in analysis. Results are consistent for vertical only and vertical plus horizontal incision patterns.

Wound dehiscence, delayed wound healing, open wounds, and wound separation are synonyms for describing a failure of primary wound healing that is not only common but that occurs in a predictable pattern. Although wound rupture occurs acutely and is associated with sudden fluctuations in local tissue tension—changes in patient position, a fall, underlying hematoma, or swelling—wound dehiscence is delayed, most typically occurring in the second surgical week.^28-30^ Wound dehiscence may be preceded by an abnormal, dusky, or grayish appearance of the wound indicative of focal tissue ischemia, and when the wound separates the margins demonstrate established marginal tissue necrosis. In breast surgery, the most typical location for wound separation is at the inferior terminus of the vertical incision where the wound tension is likely most pronounced. When the vertical incision is accompanied by a horizontal incision, the potential for dehiscence at this location may be enhanced, although interestingly in this study there was not a clinical difference between these incision patterns. Dehiscence can also occur at the upper “T junction” where the vertical incision connects with the periareolar incision, although this is less common due to lower tension levels.

**Figure 5.**
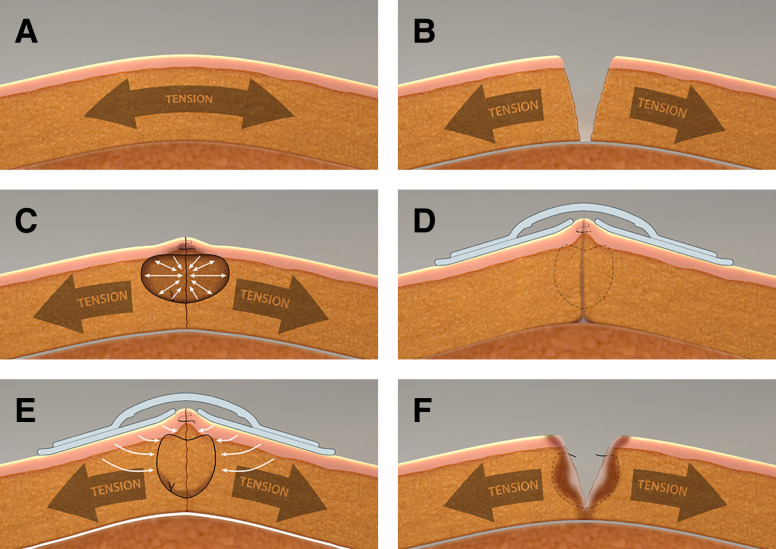
(A) The skin and subcutaneous envelope of the breast exists in a normal state of tension as part of the body's tensegrity system. This helps give structure and shape to the breast while allowing dynamic response to external forces associated with movement or applied pressure. (B) After incision, wounds separate due to release of the natural tension inherent within the system. The extent of wound separation is determined by factors such as local tension level, extent of fixation to underlying structures, tissue geometry, and tissue density. By undermining of flaps on either side of an incision, as in the development of breast flaps, the tension on either side of the wound is distributed over a greater area. (C) To approximate a wound, the tension displacing the tissues to either side must be overcome. The distracting forces are concentrated at the inner surface of the suture loops. Due to the extremely small surface area of the sutures, the adjacent focal tissue force can be markedly elevated, potentially up to 4000 mmHg. This compressive force is well above normal tissue perfusion pressures, leading to tissue ischemia. This phenomenon can be potentiated by reduction of the skin envelope and changes in volume, for example with placement of implants or as occurs secondary to swelling, which can further elevate local force levels. (D) In some instances, the ischemia secondary to elevated force inside the suture loop induces focal tissue necrosis. This loss of tissue integrity—combined with the force differential between compression intra-loop and tension extra-loop—leads to the “cheese cutting” phenomenon, in which sutures (internal or external) migrate through tissue. External sutures cause scarring manifest visually as hash mark scars. In some cases, this zone of ischemia and necrosis can be more extensive and ultimately involve the wound interface. When this occurs, adequate healing fails to occur across the wound and the wound separates as suture strength is lost. This most typically occurs at the second week after surgery. (E) Force modulating tissue bridges function by offloading tension around the wound. They can be placed as the final closure layer, or as shown in the illustration, over a final wound closure layer. Reduced lateralizing force decreases force magnitude at the tissue-suture interface, thereby decreasing the risk of ischemia and necrosis, improving the wound healing environment to decrease the risk of wound dehiscence. (F) Tension offloading with force modulating tissue bridges can be extended even as the sutures lose structural integrity, protecting the wound interface from normal tension levels. This can potentially reduce activation of tension-induced pathways leading to increased scar formation. All figures were produced by Andy Matlock.

The mechanism of postsurgical dehiscence can be informed by an exploration of local tissue characteristics, wound closure mechanism, and the physical forces present at the wound site. At a baseline our soft tissues are in a normal state of tension (Figure 5A). Tension extends through the tissue layers and varies by body area, age, genetics, anatomical location, local tissue characteristics, and with movement. Tension within the soft tissues is a vital component of our tensegrity system—the structural system of a body's structure on multiple levels and based on discontinuous rigid elements tied together (or unified) by ubiquitous tensile forces, which provides support to our bodies and governs interactions between our bodies and the external physical environment.^31^ With incision, this tensile force is released, allowing the wound margins to retract (Figure 5B). As sutures are placed and tied, they induce a compressive force (negative stress) on the tissues within the loop, whereas the tissues on the outside of the loop are in tension (positive stress) (Figure 5C). The full force of advancing the tissue to approximate the wound is concentrated at the inner surface of the suture. Because sutures are narrow with a small surface area, this force is highly concentrated, potentially exceeding 4000 mmHg, many times higher than perfusion pressure.^26,32^ This high force level can produce both focal necrosis, which inhibits normal healing, or “cheese cutting” suture migration, which may increase the forces experienced by the wound interface. Both mechanisms are contributors to wound dehiscence (Figure 5D). In breast surgery, certain factors such as tissue strength, gravitational impact of implant or breast mass, swelling, and flap design may potentiate these mechanisms, especially in the inferior pole of the breast.

FMTBs are designed to mitigate the effects of tension on healing cutaneous wounds. Based on finite element analysis, this tension offloading effect extends below the level of the dermis.^26^ The compressive forces on the inner edge of the suture are offloaded to decreased pressure-induced necrosis related to sutures (Figure 5E). In addition, the overall tension environment at the wound is relieved, potentially improving perfusion at the wound margin to promote healing. Protecting the healing zone from the effect of regional tension also minimizes the force vectors promoting suture migration (cheese cutting), both superficial and deep. In this study FMTBs proved highly effective in reducing dehiscence, likely by these mechanisms. It is also well documented that such unmitigated tension is a primary driver of poor or pathologic scar.^33,34^ With reapplication FMTBs can offload to protect the healing scar while wound strength develops (Figure 5F). This could be critical to optimizing long-term scar appearance.

Patients in this study tolerated the tissue bridges with minimal issues. With any adhesive-based device the occurrence of blisters or adhesive reactions can be a concern, but these were not encountered in this series. It should be noted that even patients with “adhesive allergies” have not had skin reactions to FMTBs in the authors’ experience. Four of 122 tissue bridge patients (3.3%) had premature cessation of therapy. With adequate degreasing with an alcohol pad, including gentle rubbing to exfoliate the attachment site, spontaneous premature detachment has not occurred in more recent patients. Because the devices are noninvasive and can be easily reapplied after surgery, if early detachment were to occur now, the authors would replace these during an office visit. In fact, long-term tissue bridge reapplication for continued mechanomodulation therapy is currently being done by some surgeons.

This study was limited by its partial retrospective nature, and limited to the first 40 FMTB patients, after which data on the intervention patients was collected prospectively. Because all patients in both patient cohorts had a final suture layer placed, this study did not directly inform wound improvement issues when the final layer of sutures is eliminated. Although this study was not sufficiently powered to assess the impact in patients with diabetes or other predisposing wound healing factors, it is conceivable that tension offloading in patients with such risks may have a positive benefit. This study did not directly address scar outcomes, and a separate randomized controlled trial is currently underway to evaluate scar outcomes with extended wear or reapplication of FMTBs. Another limitation of this study was that the exact duration of adherence of the FMTBs was not recorded in this study, however it was noted that a single application of FMTBs would typically stay in place for 3 or more weeks in this anatomical location. This study also had the potential impact of conflict of interest of 2 of the authors. To mitigate this factor, all initial chart reviews and data collation were completed solely by coauthors who did not have any conflict of interest related to Brijjit Medical, Inc., which was the manufacturer of the devices. Similarly, the authors with conflicts did not participate in the statistical analyses.

## CONCLUSIONS

FMTBs are effective for tension offloading of at-risk breast incision wounds, both at the time of wound closure and over the treatment time. This effect is associated with a marked decrease in wound dehiscence, and with extended wear through reapplication may lead to better scar outcomes.

## Supplemental Material

This article contains supplemental material located online at www.aestheticsurgeryjournal.com.

## Supplementary Material

sjad285_Supplementary_Data
